# Supplementary material for: Structure of human lysosomal acid α-glucosidase–a guide for the treatment of Pompe disease
Source: Nat Commun. 2017 Oct 24;8:1111. doi: 10.1038/s41467-017-01263-3 (PMC5653652; doi:10.1038/s41467-017-01263-3)
Supplement: Supplementary file 1 — Supplementary information [file 41467_2017_1263_MOESM1_ESM.pdf]

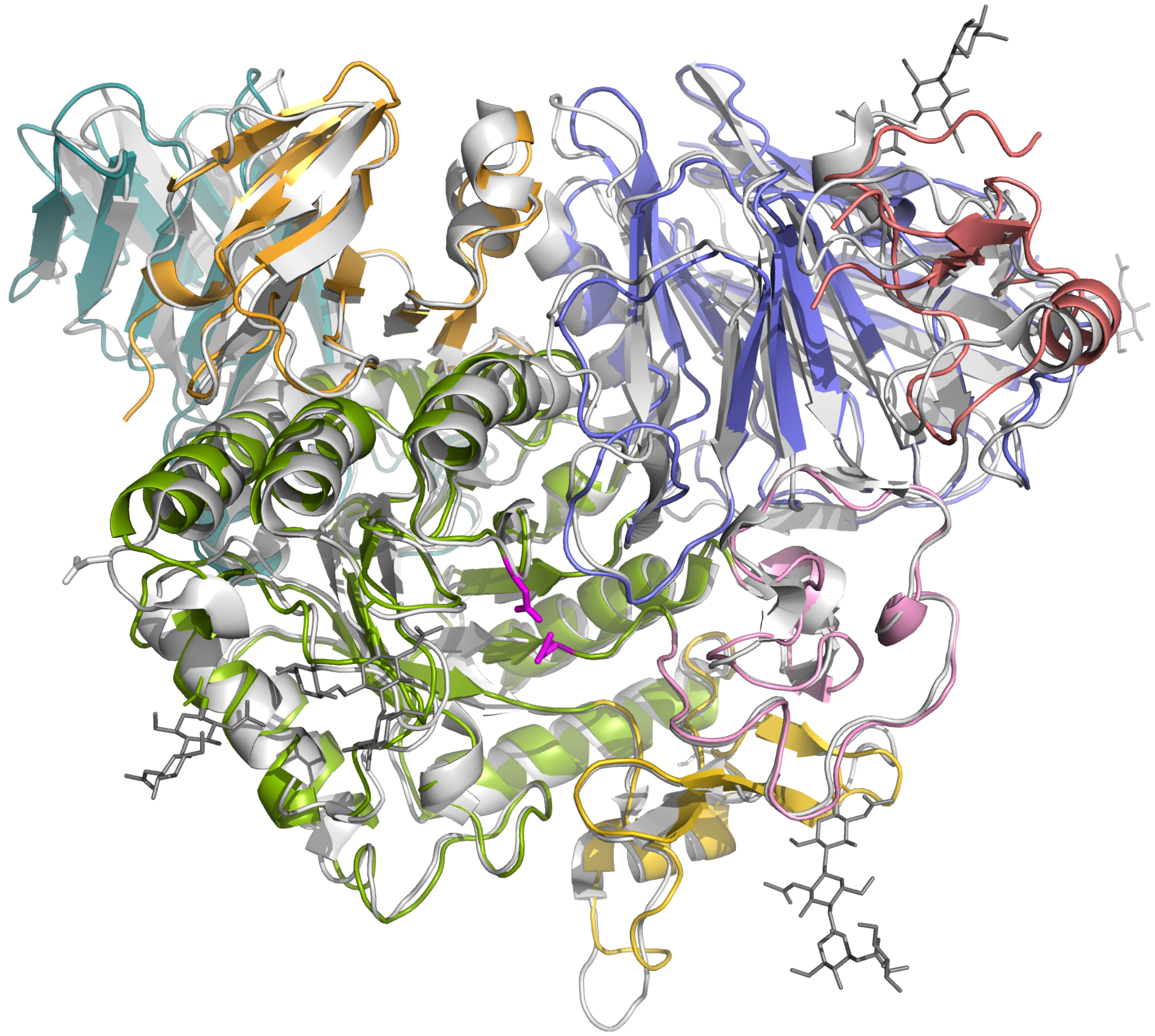

Supplementary Figure 1 | Comparison of the overall structures of GH31 members rhGAA and NtMGAM. Cartoon representation of the structure of rhGAA, colour coded as in **Fig. 2c**, overlaid onto a cartoon representation of the structure of NtMGAM in grey (PDB ID 2QLY). The glycan chains present on rhGAA are depicted as sticks in grey.

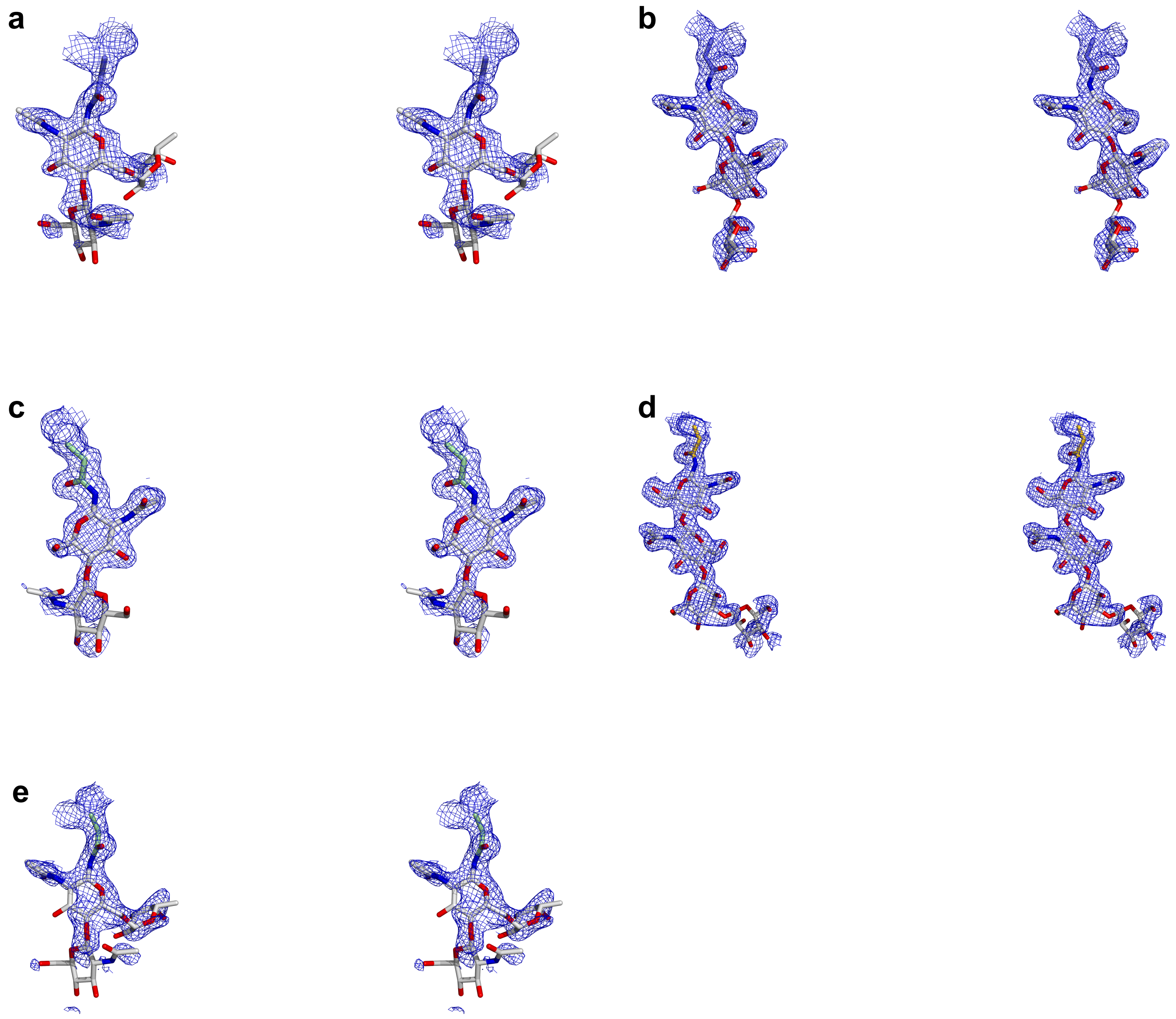

Supplementary Figure 2 | Stereoimages of glycan chains decorating the rhGAA structure. Stick representation of glycan structures and the Asn residues they are attached to with weighted *2Fo-Fc* electron density maps contoured at 1.0 σ shown in blue. **a**, Glycan chain bound to N140. **b**, Glycan chain bound to N233. **c**, Glycan chain bound to N390. **d**, Glycan chain bound to N470. **e**, Glycan chain bound to N652.

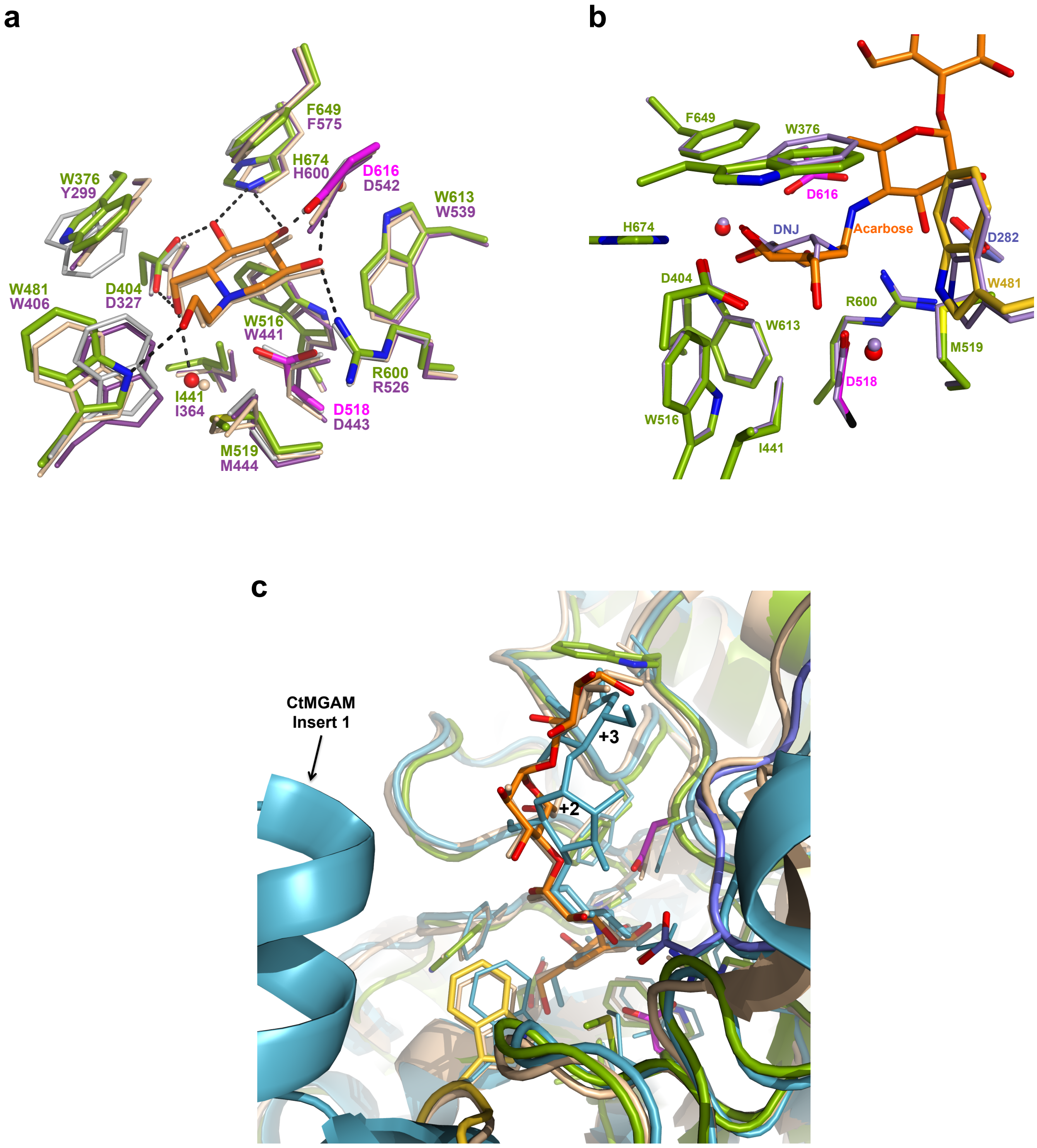

Supplementary Figure 3 | Comparison of ligand binding to rhGAA, NtMGAM and CtMGAM and substrate specificity of rhGAA. a, Overlap of active site residues binding NHE-DNJ (orange) in rhGAA (green and magenta carbon atoms) with active site residues binding NHE-DNJ in NtMGAM (wheat) (PDB ID 3L4W). Due to the bulky hydroxyethyl N-substitution on NHE-DNJ, in both complexes the Trp and Met residues corresponding to rhGAA positions 481 and 519, respectively, move away from the active site with respect to unbound rhGAA (grey) and unbound NtMGAM (purple). **b**, Overlap of the DNJ-rhGAA complex (purple sticks) onto acarbose (orange) bound to rhGAA (colour coded as in **Fig. 2c**). **c**, Acarbose binding to rhGAA (colour coded as in **Fig. 2c**) compared to acarbose binding to NtMGAM in wheat (PDB ID 2QMJ) and the C-terminal domain of MGAM (CtMGAM) in steelblue (PDB ID 3TOP). Despite the paucity of contacts in substrate binding subsites +2 and +3, the overall pose of acarbose bound to rhGAA is identical to the one of acarbose bound to NtMGAM. Insert I of CtMGAM contains an additional α-helix of 21 residues, which provides supplementary hydrogen bonds to the sugar units in subsite +2 and +3[1], accounting for the preference of CtMGAM for longer substrates and a different pose of acarbose as compared to the one adopted in rhGAA and NtMGAM.

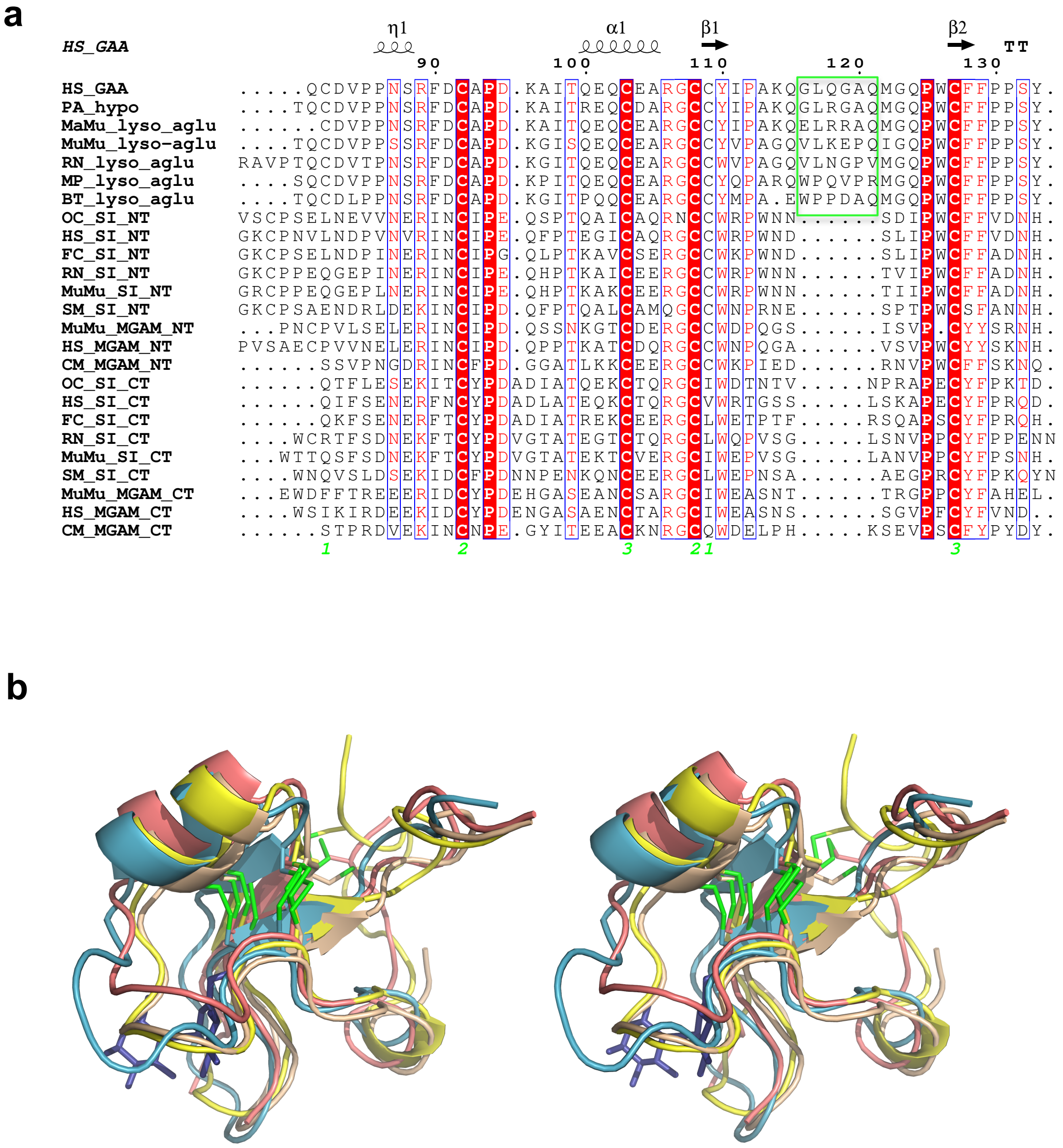

Supplementary Figure 4 | The trefoil type-P domain. **a**, Sequence alignment of trefoil type-P domains of members of glycoside hydrolase family GH31. NCBI Genbank accession numbers are provided in parentheses and N-terminal and C-terminal domain are indicated were appropriate. *Homo sapiens* GAA (AAA52506.1), *Pongo abelii* hypothetical protein (CAH92351.1), *Macaca mulatta* lysosomal α-glucosidase (AFE67011.1), *Mus musculus* lysosomal α-glucosidase (AAB06943), *Rattus norvegicus* acidic α-glucosidase (AAH61753.1), *Mustela putorius furo* acid α-glucosidase (AER98854.1), *Bos taurus* acidic α-glucosidase (AAF81636.1), *Oryctolagus cuniculus* sucrase-isomaltase (AAA31459.1), *Homo sapiens* sucrase-isomaltase (AAI15035.1), *Felis catus* sucrase-isomaltase (BAG16411.1), *Rattus norvegicus* sucrase-isomaltase (AAA65097.1), *Mus musculus* 129/Sv sucrase-isomaltase (ACH86012.1), *Suncus murinus* sucrase-isomaltase (BAA25370.1), *Mus musculus* maltase-glucoamylase (ACH86011.1), *Homo sapiens* maltase-glucoamylase (AAC39568.2), *Callorhinchus milii* maltase-glucoamylase (AFO93797.1). The sequence segment unique to the lysosomal representatives of family GH31, and removed during maturation of GAA[2], is boxed in green. Secondary structure elements are shown at the top of the alignment and cysteine residues involved in disulphide bridge formation are marked in green at the bottom of the alignment. **b**, Stereoimage of a structural overlap of the trefoil type-

P domains of rhGAA in salmon, NtMGAM (PDB ID 2QLY) in wheat, CtMGAM (PDB ID 3TON) in steelblue and the N-terminal domain of SI, NtSI (PDB ID 3LPO), in yellow. The rhGAA trefoil Type-P domain is stabilized by three disulphide bridges, two of which are conserved amongst the human intestinal homologues. The disulphide bond corresponding to rhGAA C82-C109 is not conserved in CtMGAM, where the residue corresponding to rhGAA C82 is replaced by an Ile residue, and in the structure of NtMGAM, where it could not be modelled because of disorder of the N-terminal region[3]. The acarvosine moiety bound to rhGAA is depicted in blue sticks.

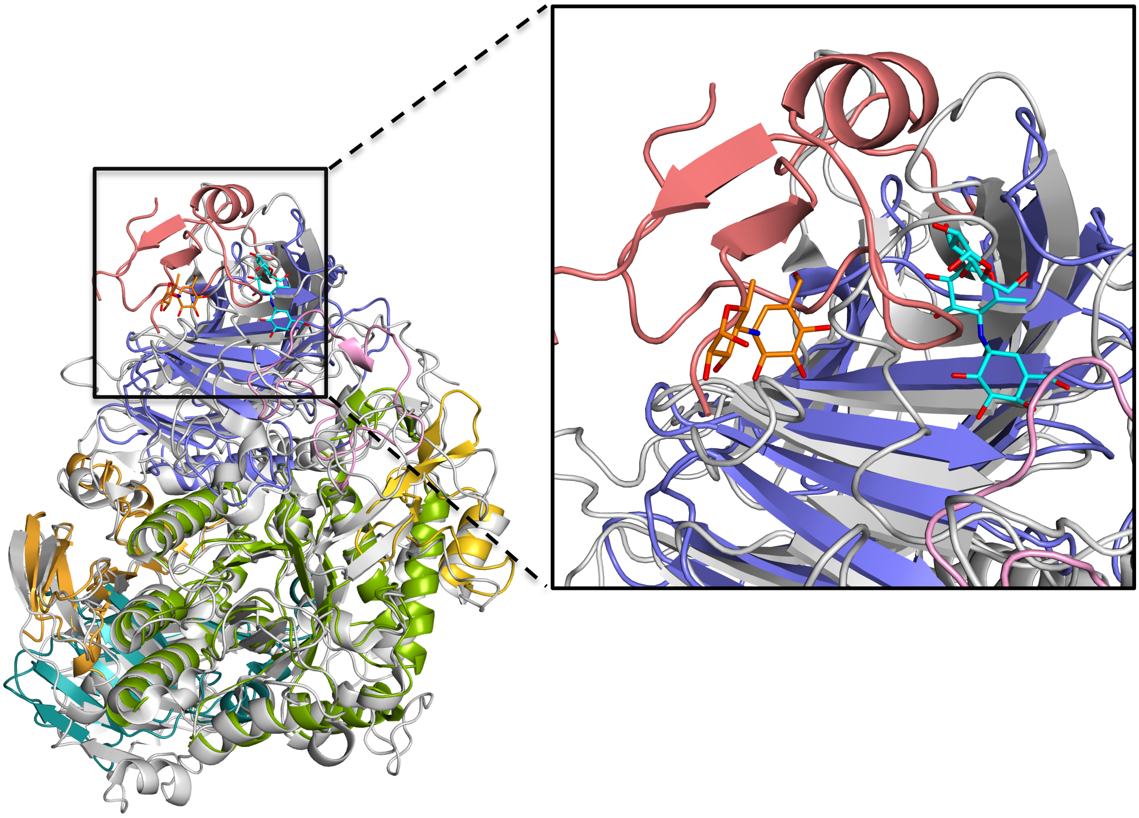

Supplementary Figure 5 | Overall location of the secondary substrate-binding domain. Cartoon overlap of the rhGAA-acarbose complex, colour coded as in **Fig. 2c**, and the acarbose complex of *Gracilariopsis lemaneiformis* α-1,4-glucan lyase, GLase (PDB ID 2X2I), colour coded in grey. Carbon atoms of the acarvosine unit bound to rhGAA are coloured in orange and carbon atoms of the trisaccharide derived from acarbose bound to α-1,4-glucan lyase are colour coded in cyan. The insert shows a close-up view. GLase is devoid of a trefoil type-P domain (salmon) and the trisaccharide is bound to the N-terminal β-sheet domain.

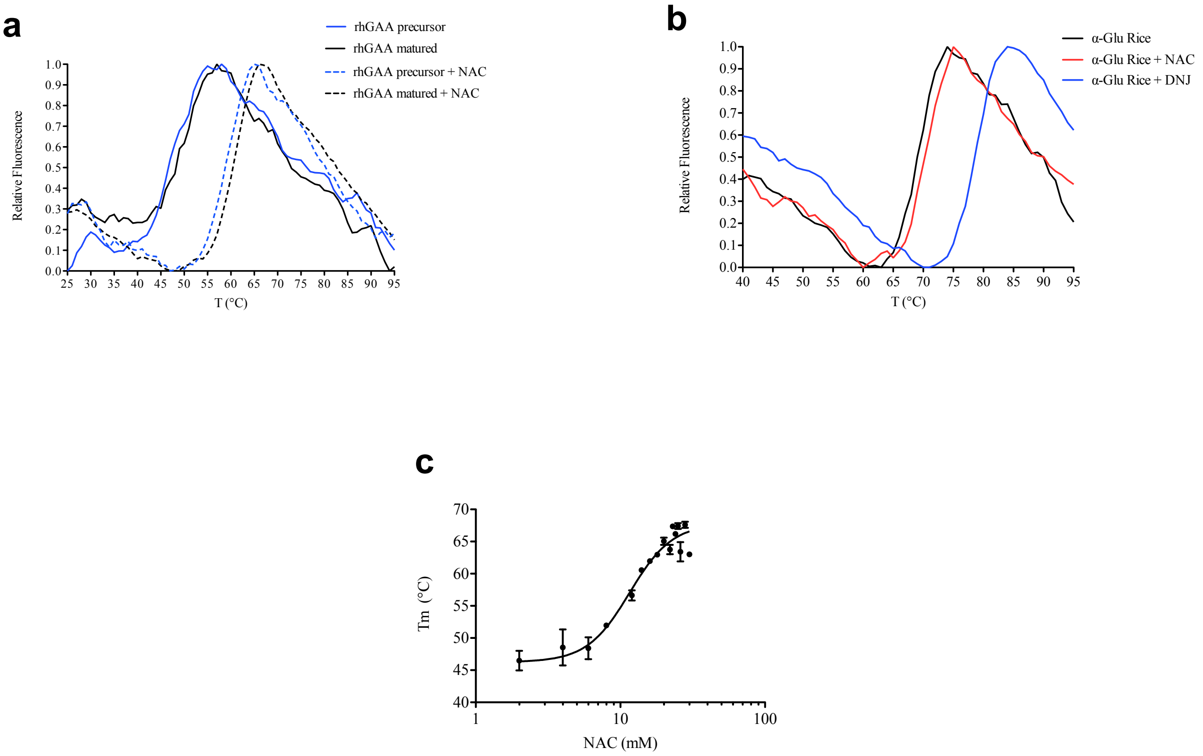

Supplementary Figure 6 | Analysis of the effect of pharmacological chaperones. a, Thermal scans of the rhGAA precursor and matured forms in presence of the pharmacological chaperone NAC. **b**, Thermal scans of α-glucosidase from rice in presence of the pharmacological chaperones NAC and DNJ. **c**, Determination of the interaction rhGAA-NAC by differential scanning fluorimetry. Thermal scans were performed in triplicate and fluorescence was normalized to the maximum value within each scan to obtain relative fluorescence. Melting temperatures were calculated according to Niesen *et al.*, 2007[4]. The standard deviations for each melting temperature were calculated from three replicates. For the determination of the dissociation constant (K_D) of NAC experimental data were best fitted according to a simple cooperative model equation reported in Vivoli *et al.,* 2014[5].

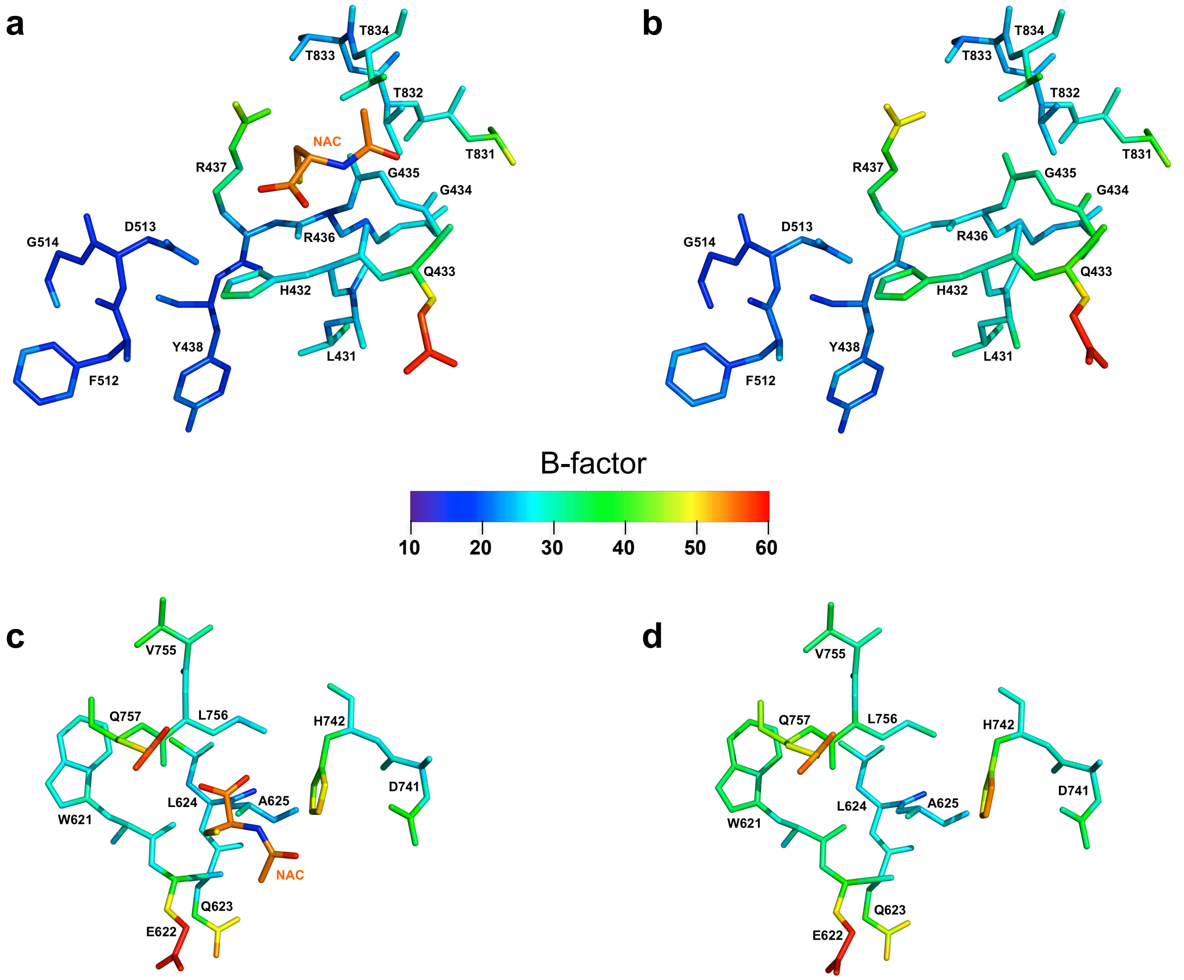

Supplementary Figure 7 | The stabilizing function of NAC. Stick representation of residues located in the vicinity of the NAC binding sites colour coded by B-factor variance. Carbon atoms of NAC are represented in orange. **a**, Fully occupied NAC1 binding site in the rhGAA-NAC complex. **b**, Region corresponding to **a**, in the unbound rhGAA structure. **c**, Partially occupied NAC2 binding site in the rhGAA-NAC complex. **d,** Region corresponding to **c**, in the unbound rhGAA structure. The B-factors of unbound rhGAA have been normalized by subtraction of 7.45 $Å^2$ from each individual atomic B-factor according to the overall B-factors difference as outlined in **Table 1**.

a

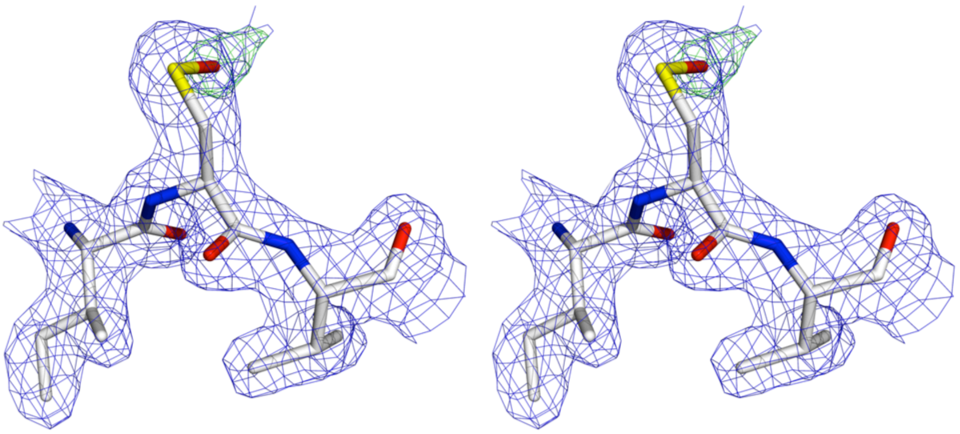

b

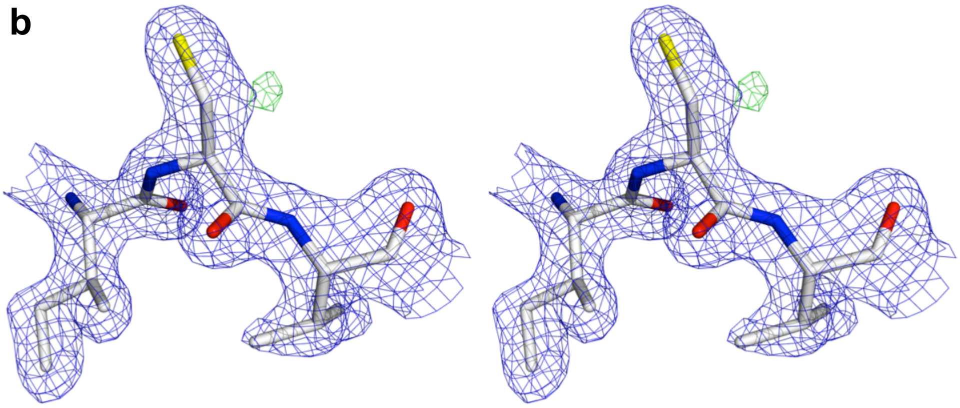

Supplementary Figure 8 | The anti-oxidative effect of NAC. a, C938 is oxidized to the sulfenic acid form upon exposure to X-rays, except for the **b**, rhGAA-NAC complex, where NAC exercised an anti-oxidative effect. Stereoimage of stick representation of residues I937, C938 and V939 from rhGAA and from the rhGAA-NAC complex with weighted *2Fo-Fc* electron density maps, contoured at 1.5 σ, shown in blue and *Fo-Fc* difference electron density maps (calculated for models prior modification of C938 to the sulfenic acid form) contoured at 4.0 σ shown in green.

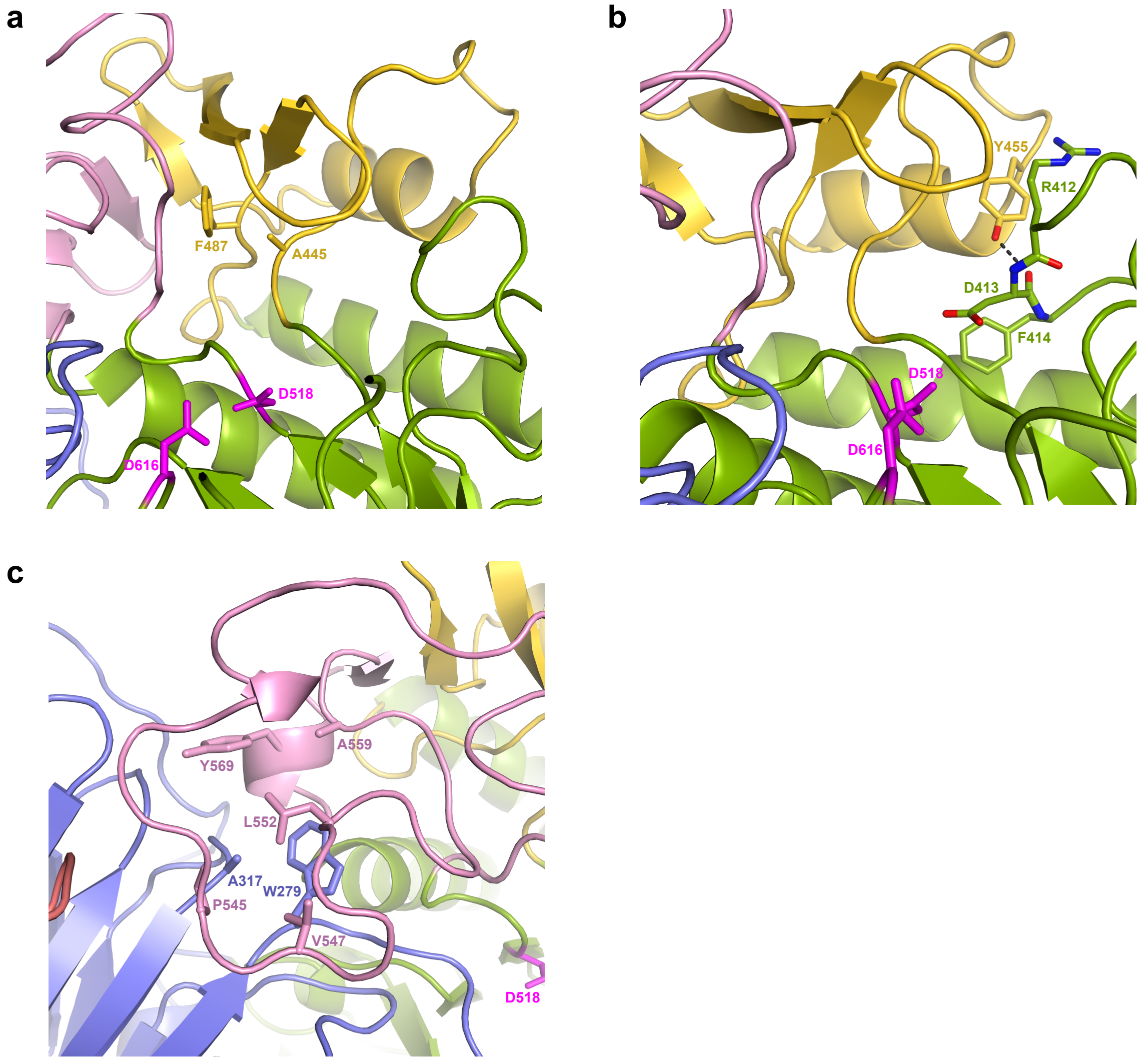

Supplementary Figure 9 | GAA mutants responsive to NAC. Cartoon representation of rhGAA colour coded as in **Fig. 2c**, with selected residues shown as sticks. **a**, A445 and F487 are located at the boundaries of insert I. The A445P mutant, where insert I is probably destabilized, is responsive to NAC. **b**, Y455 stabilizes *via* a hydrogen bond a long surface loop in the vicinity of the active site. The Y455F mutant, where this contact is abolished, is responsive to NAC. **c**, L552 on insert II establishes hydrophobic contacts with the N-terminal β-sheet domain. The L552P mutant, where this interaction is probably perturbed, is responsive to NAC.

Supplementary Table 1 | GAA missense mutations associated with Pompe disease

Mutation	ASA[*] %	B/E[#]	Structural consequences	Ref.
C103G<br>C103R	0	B	loss of stabilizing disulfide bridge and/or severe structure perturbation due to steric clash caused by the introduction of bulky side-chain	6<br>7
C108G	7	B	loss of stabilizing disulfide bridge	7
C127F	3	B	loss of stabilizing disulfide bridge and severe structure perturbation due to steric clash caused by the introduction of bulky side-chain	7
L141M	41	E	steric clash due to increased size of side-chain and destabilization of hydrophobic core	8
R154P	3	B	loss of large basic side-chain making stabilizing H-bonds with three main-chain carbonyl groups; main-chain geometry distortion and steric clash caused by introduction of proline	9
R168Q	43	E	loss of large basic side-chain establishing stabilizing H-bonds with acidic side-chain - altered H-bonding network	10
L169P	0	B	β-strand main-chain torsion angles incompatible with proline geometry	11
R178H	20	B	loss of large basic side-chain establishing stabilizing H-bond with acidic side-chain	12
R190H	12	B	loss of large basic side-chain making stabilizing H-bonds with acidic side-chain and two main-chain carbonyl groups	13
Y191C	14	B	loss of large side-chain participating in stabilizing H-bond and inter-domain π-stacking interactions	14
L208P	25	B	main-chain torsion angle distortion and loss of stabilizing H-bond between main-chain nitrogen and carbonyl oxygen of neighbouring loop	15
P217L	18	B	loss of proline-induced main-chain geometry in a coil	16
G219R	0	B	disruption of hydrophobic core due to severe steric clash caused by the introduction of bulky side-chain and loss of conformational freedom of main-chain torsion angles	17
V222M	0	B	steric clash due to increased size of side-chain and destabilization of hydrophobic core	18
R224P<br>R224Q<br>R224W	4	B	main-chain torsion angle distortion, loss of basic residue involved in stabilizing H-bonding interactions with an acidic side-chain and two main-chain carbonyl groups, and structure perturbation caused by steric clash due to introduction of bulky side-chain	19<br>20<br>21
T234K<br>T234R	3	B	loss of stabilizing H-bond and severe perturbation of hydrophobic core due to introduction of bulky polar side-chains	22<br>7
A237V	8	B	steric clash due to increased size of side-chain within hydrophobic core	23
A242V	1	B	steric clash due to increased size of side-chain within hydrophobic core	13
L246R	0	B	severe steric clash and disruption of hydrophobic core by introduction of bulky polar side-chain	24
L248P	5	B	β-strand main-chain torsion angles incompatible with proline geometry; loss of β-sheet stabilizing H-bond	13
G259V	2	B	loss of conformational freedom of main-chain torsion angles and steric clash due to introduction of side-chain within hydrophobic core	13
E262K	0	B	steric clash and disruption of stabilizing H-bonding network established between N-terminal β-sheet domain and catalytic domain by substitution of acidic side-chain by larger and basic side-chain	17
P266S	58	E	loss of proline-induced main-chain geometry in a coil	25
T271A	8	B	loss of stabilizing H-bond	19

R281W	15	B	loss of basic residue involved in stabilizing H-bonding interaction with a main-chain carbonyl group of the catalytic domain and structure perturbation caused by steric clash due to introduction of bulky side-chain	18
P285R<br>P285S	3	B	loss of proline-induced main-chain geometry in a coil, severe steric clash with catalytic domain due to introduction of a bulky side-chain and/or perturbation of hydrophobic core by introduction of polar side-chains	6<br>13
L291F<br>L291H<br>L291P	0	B	disruption of hydrophobic core by introduction of bulky or polar side-chain; main-chain geometry distortion by introduction of proline	13<br>13<br>13
Y292C	0	B	loss of large side-chain participating in stabilizing π-stacking interactions with catalytic domain	26
G293R	1	B	structure perturbation owed to severe steric clash with catalytic domain due to the introduction of bulky side-chain and loss of conformational freedom of main-chain torsion angles	6
H295Q	0	B	loss of stabilizing H-bond between N-terminal β-sheet domain and catalytic domain	27
Y298S	0	B	loss of large side-chain participating in stabilizing π-stacking interactions and introduction of hydroxyl group into hydrophobic environment	8
L299R<br>L299P	1	B	severe disruption of hydrophobic core by introduction of bulky and polar side-chain or main-chain geometry distortion by introduction of proline	28<br>13
S306L	27	B	surface-exposure of hydrophobic residue	29
H308L<br>H308P	1	B	loss of side-chain participating in stabilizing H-bond and π-stacking interactions or β-strand main-chain torsion angles incompatible with proline geometry	15<br>6
G309R	0	B	structure disruption caused by severe steric clash due to the introduction of bulky side-chain and loss of conformational freedom of main-chain torsion angles	30
L312R	1	B	severe disruption of hydrophobic core by introduction of bulky and polar side-chain	6
N316I	1	B	loss of stabilizing H-bond between N-terminal β-sheet domain and insert II and introduction of hydrophobic side-chain into polar environment	7
M318K<br>M318T	0	B	perturbation of hydrophobic environment by introduction of polar side-chains	13<br>31
P324L	19	B	loss of correct main-chain geometry in proline-rich coil	15
W330G	3	B	loss of large side-chain participating in stabilizing π-stacking interactions within hydrophobic core	32
G334S	3	B	structure perturbation caused by steric clash and loss of conformational freedom of main-chain torsion angles	33
G335E<br>G335R	5	B	severe structure perturbation caused by steric clash due to the introduction of bulky side-chains and loss of conformational freedom of main-chain torsion angles	7<br>13
P347R	0	B	severe disruption of hydrophobic core by introduction of bulky and polar side-chain, steric clash with proximal β-sheet domain and loss of proline-imposed structural rigidity of succeeding α-helix	13
V350M	0	B	severe steric clash due to increased size of side-chain and destabilization of hydrophobic core	18
L355P	1	B	disruption of α-helical structure by helix-breaking proline; loss of stabilizing van der Waals interactions with catalytic domain	6
P361L	1	B	severe steric clash and loss of correct main-chain geometry in proline-rich coil	34
W367R	2	B	structure perturbation owed to steric clash and loss of extensive stabilizing π-stacking interactions	35

**L369P**	0	B	perturbation of hydrophobic core due to larger side-chain and disruption of α-helical structure by helix-breaking proline	36
**H372L**	0	B	loss of stabilizing H-bonding interactions	37
**L373R**	0	B	severe disruption of hydrophobic core due to introduction of bulky and polar side-chain	29
**C374R**	1	B	structure perturbation owed to severe steric clash due to introduction of bulky side-chain	6
**R375L**<br>**R375H**	11	B	loss of large basic side-chain establishing H-bonds with three carbonyl groups stabilizing a loop close to the active site	14<br>38
**G377R**	28	B	loss of conformational freedom of main-chain torsion angles in loop	39
**P397L**	3	B	disruption of hydrophobic core due to larger side-chain and loss of well conserved proline residue imposing main-chain geometry in coil	7
**Q401R**	0	B	structure perturbation owed to severe steric clash due to introduction of bulky side-chain	14
**D404N**	4	B	loss of well conserved acidic residue interacting with substrate in substrate-binding subsite -1	40
**L405P**	3	B	disruption of active site by main-chain geometry distortion due to introduction of proline	6
**D406N**	16	B	loss of H-bonding interaction with main-chain nitrogen within a loop participating in active site architecture	18
**M408I**<br>**M408V**	3	B	loss of π-stacking interactions and perturbation of hydrophobic core near the active site	41<br>17
**D413E**	2	B	steric clash due to introduction of larger side-chain leading to perturbation of the active site	42
**D419V**	84	E	surface-exposure of hydrophobic residue	7
**R437C**	15	B	loss of side-chain participating in stabilizing H-bond and π-stacking interactions	34
**M439K**	0	B	disruption of hydrophobic core by introduction of polar side-chain	25
**V442M**	2	B	steric clash due to increased size of side-chain and destabilization of hydrophobic core	12
**A445P**	7	B	main-chain geometry distortion due to introduction of proline and destabilization of insert I	43
**Y455F**<br>**Y455C**	5	B	loss of H-bond and/or π-stacking interactions stabilizing a long loop contributing to active site architecture	6<br>11
**P457H**<br>**P457L**	6	B	disruption of hydrophobic core due to the introduction of larger and polar side-chain and loss of proline-imposed structural rigidity of following α-helix	7<br>13
**D459N**	25	B	perturbation of complex hydrogen-bonding network involving the Asp side-chain and two main-chain nitrogens of an adjacent surface loop stabilizing the overall scaffold of insert I	8
**G461S**	0	B	perturbation of hydrophobic core due to introduction of polar side-chain, steric clash and loss of conformational freedom of main-chain torsion angles	13
**V466G**<br>**V466F**	0	B	loss of stabilizing van der Waals interactions and too much conformational freedom of main-chain torsion angles or disruption of hydrophobic core due to steric clashes caused by the introduction of a bulky side-chain	35<br>13
**N470T**	16	B	loss of glycosylation site affecting enzyme processing and trafficking and loss of H-bonding interaction within surface loop stabilizing insert I	18
**G478R**	0	B	severe structure perturbation caused by steric clash due to the introduction of bulky side-chain and loss of conformational freedom of main-chain torsion angles	44

**K479N**	48	E	apparently harmless substitution of polar by polar side-chain on the protein surface	8
**W481R**	29	B	loss of stabilizing van der Waals interactions adjacent to substrate-binding subsite -1 and steric hindrance to substrate binding due to longer side-chain	45
**P482R**<br>**P482L**	25	B	severe structure perturbation owed to steric clash introduced by larger side-chains and loss of proline-induced main-chain geometry within a coil structuring the active site	13<br>11
**G483V**<br>**G483R**	27	B	loss of conformational freedom of main-chain torsion angles within a coil structuring the active site and/or surface-exposure of a hydrophobic residue	13<br>13
**A486P**	0	B	β-strand main-chain torsion angles incompatible with proline geometry and introduction of steric clashes	16
**F487S**	0	B	disruption of hydrophobic core due to loss of stabilizing van der Waals interactions and introduction of a polar side-chain	13
**D489G**<br>**D489N**	3	B	perturbation or loss of complex stabilizing hydrogen-bonding network involving the well conserved Asp side-chain and main- and side-chain atoms of surface loops anchoring insert I onto insert II	29<br>43
**F490L**	3	B	loss of stabilizing π-stacking and van der Waals interactions within the hydrophobic core	18
**W499R**	0	B	loss of stabilizing van der Waals interactions and perturbation of hydrophobic core due to introduction of long and polar side-chain	27
**G514R**	0	B	severe structure perturbation caused by steric clash due to the introduction of bulky side-chain and loss of conformational freedom of main-chain torsion angles	46
**M515K**	1	B	disruption of hydrophobic core by introduction of polar side-chain	47
**M519T**<br>**M519V**	3	B	loss of stabilizing π-stacking and van der Waals interactions adjacent to substrate-binding subsite -1	44<br>48
**E521K**<br>**E521Q**	1	B	loss well-conserved Glu involved in complex hydrogen-bonding network stabilizing the active site architecture	49<br>13
**P522A**<br>**P522S**<br>**P522T**	0	B	perturbation of hydrophobic core due to the introduction of larger and polar side-chains and/or loss of proline-induced main-chain geometry within a coil structuring the active site	50<br>13<br>46
**S523Y**	2	B	loss of stabilizing H-bond to Asp282 from the N-terminal β-sheet domain involved in substrate binding and steric hindrance to substrate binding in subsite +1 due to introduction of longer side-chain	7
**F525Y**	37	B	perturbation of active site architecture by loss of stabilizing van der Waals interactions due to the introduction of a polar group	19
**P545L**	3	B	steric clash due to the introduction of larger side-chain and loss of proline-induced main-chain geometry within proline-rich coil in insert II	51
**G546R**	18	B	severe steric clash with portions of the N-terminal β-sheet domain due to the introduction of bulky side-chain and loss of conformational freedom of main-chain torsion angles within proline-rich coil in insert II	52
**G549R**	69	E	loss of conformational freedom of main-chain torsion angles within proline-rich coil in insert II	6
**L552P**	1	B	main-chain geometry distortion of coil in insert II due to introduction of proline and loss of stabilizing van der Waals interactions between insert II and the N-terminal β-sheet domain	53
**T556A**	1	B	loss of H-bond stabilizing long coil in insert II	54
**I557F**	0	B	destabilization of hydrophobic core caused by steric clashes due to introduction of bulky side-chain	19

**C558S**	4	B	loss of stabilizing disulfide bridge	55
**S566P**	21	B	loss of H-bond stabilizing insert II and main-chain geometry distortion of coil due to introduction of proline	56
**H568L**	0	B	loss of stabilizing H-bonding network and introduction of hydrophobic residue into polar environment	57
**N570K**	12	B	disruption of stabilizing H-bonding network	13
**H572Q**	0	B	loss of stabilizing H-bonds	13
**N573K**	1	B	loss of well conserved Asn involved in stabilizing H-bonding interaction and formation of steric clashes due to introduction of larger side-chains	29
**N573H**				13
**Y575C**	1	B	loss of large side-chain participating in stabilizing H-bond and π-stacking interactions	7
**Y575S**				6
**G576R**	0	B	severe structure disruption owed to steric clashes due to the introduction of more or less bulky side-chains and loss of conformational freedom of main-chain torsion angles required for termination of preceding α-helix	29
**G576S**				58
**G576D**				13
**E579K**	0	B	apparently harmless substitution leading to the formation of a favourable H-bonding interaction	6
**S583F**	0	B	loss of stabilizing H-bond and disruption of adjacent hydrophobic core due to introduction of bulky side-chain	13
**R585K**	46	E	apparently harmless substitution at the protein surface, apart from surface-exposure of hydrophobic methionine	13
**R585M**				15
**R594H**	6	B	loss of large basic side-chain establishing inter-domain stabilizing H-bonds with two main-chain carbonyl groups and two acidic side-chains; main-chain geometry distortion and steric clash caused by introduction of proline	7
**R594P**				13
**S599Y**	0	B	severe overall structure perturbation caused by steric clashes due to introduction of bulky side-chain	14
**R600C**	2	B	loss of conserved residue structuring the active site and responsible for substrate binding in subsite +1	59
**R600H**				60
**R600L**				46
**S601L**	0	B	disruption of active site architecture by loss of stabilizing H-bonding network and/or by steric clash due to introduction of bulky side-chain	29
**S601W**				35
**T602A**	0	B	loss of stabilizing H-bond within the β-barrel of the catalytic domain	7
**G605D**	7	B	loss of conformational freedom of main-chain torsion angles required for initiation of α-helix and steric clash with N-terminal domain due to introduction of side-chain (conserved Gly)	61
**G607D**	2	B	loss of conformational freedom of main-chain torsion angles and severe structure perturbation by steric clash due to introduction of side-chain	6
**A610V**	2	B	severe structure perturbation by steric clash due to introduction of larger side-chain	50
**G611D**	0	B	loss of conformational freedom of main-chain torsion angles and severe structure perturbation by steric clash due to introduction of side-chain	29
**H612Q**	0	B	loss of stabilizing H-bonding network or disruption of $(\beta/\alpha)_8$-barrel core by introduction of larger side-chain	43
**H612Y**				16
**T614K**	1	B	structure disruption near the active site by steric clash due to introduction of long side-chain	13

**G615R**	0	B	severe disruption of active site architecture by steric clashes due to the introduction of bulky side-chains and loss of conformational freedom of main-chain torsion angles (conserved Gly)	60
**G615E**				57
**D616N**	22	B	loss of catalytic acid/base	10
**S619R**	13	B	loss of stabilizing H-bonding network and structure perturbation by steric clashes due to introduction of more or less bulky side-chains	58
**S619N**				62
**S627P**	3	B	loss of stabilizing H-bonding network and disruption of α-helical structure by helix-breaking proline	7
**N635K**	1	B	disruption of complex stabilizing H-bonding network established between the catalytic domain and the proximal β-sheet domain	16
**N635D**				18
**G638V**	2	B	severe structure disruption owed to steric clashes with N-terminal β-sheet domain due to the introduction of more or less bulky side-chains and loss of conformational freedom of main-chain torsion angles required for termination of preceding α-helix (conserved Gly)	37
**G638W**				63
**V642D**	0	B	disruption of hydrophobic core by introduction of charged side-chain	64
**G643R**	0	B	severe structure disruption by steric clash due to introduction of bulky side-chain and loss of conformational freedom of main-chain torsion angles	65
**A644P**	3	B	main-chain geometry distortion by introduction of proline	11
**D645N**	0	B	loss of strictly conserved residue helping to forge the active site architecture	66
**D645E**				67
**D645H**				68
**D645Y**				11
**C647W**	0	B	loss of stabilizing disulfide bridge and severe structure perturbation due to steric clash caused by the introduction of bulky side-chain	69
**G648D**	0	B	severe structure disruption by steric clashes due to introduction of side-chains and loss of conformational freedom of main-chain torsion angles (conserved Gly)	70
**G648S**				66
**T653N**	17	B	structure disruption by steric clash due to introduction of longer side-chain	20
**S654P**	44	E	main-chain geometry distortion by introduction of proline	8
**R660H**	1	B	loss of well-conserved basic residue establishing stabilizing H-bonds with carbonyl groups of the proximal β-sheet domain	58
**R660C**				35
**W661G**	0	B	loss of large side-chain participating in stabilizing H-bond and extensive π-stacking interactions	13
**M671R**	0	B	severe disruption of hydrophobic core by introduction of bulky and charged side-chain	39
**R672Q**	0	B	loss of well-conserved basic side-chain participating in stabilizing salt-bridge and π-stacking interactions and/or structure perturbation by steric clash due to the introduction of bulky side-chain	66
**R672W**				66
**E689K**	53	E	apparently harmless substitution of charges on the protein surface	71
**R702C**	1	B	loss of well-conserved basic residue establishing a complex stabilizing H-bonding network with four main-chain carbonyl groups from different structural elements	72
**R702H**				73
**R702L**				29
**T711R**	3	B	loss of stabilizing inter-domain H-bond and structure disruption by steric clash with the distal β-sheet domain due to introduction of bulky side-chain	73

**L712P**	11	B	disruption of α-helical structure by helix-breaking proline	13
**V723M**	0	B	disruption of hydrophobic core between catalytic and proximal β-sheet domain by introduction of larger side-chain	73
**A724D**	0	B	disruption of hydrophobic core between catalytic and proximal β-sheet domain by introduction of larger and charged side-chain	74
**R725Q**<br>**R725P**<br>**R725W**	1	B	loss of basic residue establishing a complex stabilizing salt-bridge and H-bonding network with acidic and polar side-chains and a main-chain carbonyl group; main-chain geometry distortion by introduction of proline; structure perturbation by steric clash due to the introduction of bulky side-chain	18<br>8<br>65
**L729R**	1	B	disruption of hydrophobic core between proximal β-sheet domain, catalytic and N-terminal β-sheet domain by introduction of large and polar side-chain	75
**T737N**	0	B	perturbation of stabilizing H-bonding network and structure disruption by steric clash due to introduction of longer side-chain	13
**Q743K**<br>**Q743R**	3	B	perturbation of stabilizing H-bonding network and structure disruption by steric clash due to introduction of longer side-chains	7<br>42
**W746G**<br>**W746S**<br>**W746R**<br>**W746C**<br>**W746L**	0	B	loss of stabilizing H-bond and van der Waals interaction in the hydrophobic core between the proximal β-sheet and the catalytic domain and/or disruption of the hydrophobic core by introduction of polar or charged residues	19<br>13<br>36<br>8<br>18
**G759A**	72	E	loss of conformational freedom of main-chain torsion angles in surface loop	76
**E762K**	60	E	apparently harmless substitution of charges on the protein surface	10
**Y766S**<br>**Y766C**	2	B	loss of large side-chain participating in stabilizing H-bond and π-stacking interactions	77<br>78
**P768R**	1	B	severe structure perturbation by steric clash due to the introduction of bulky and polar side-chain into a hydrophobic environment and loss of proline-induced main-chain geometry	44
**W772R**	0	B	disruption of hydrophobic core by introduction of charged side-chain and loss of stabilizing van der Waals and π-stacking interactions	79
**D774N**	18	B	apparently harmless substitution at the protein surface	80
**H799Y**	66	E	apparently harmless substitution at the protein surface	80
**R819P**	20	B	β-strand main-chain torsion angles incompatible with proline geometry, introduction of steric clash disrupting β-sheet structure and loss of stabilizing H-bonding interactions	29
**L843P**	0	B	β-strand main-chain torsion angles incompatible with proline geometry, introduction of steric clash disrupting β-sheet structure and loss of stabilizing van der Waals interactions	29
**A880D**	2	B	perturbation of hydrophobic environment by introduction of charged side-chain	81
**L901Q**	1	B	severe perturbation of hydrophobic environment by introduction of polar and larger side-chain	82
**V916F**	2	B	severe disruption of hydrophobic environment by introduction of bulky side-chain	7
**Y928C**	19	B	loss of stabilizing H-bonding interactions	29
**L935P**	0	B	β-strand main-chain torsion angles incompatible with proline geometry, introduction of steric clash disrupting β-sheet structure and loss of stabilizing van der Waals interactions	13

**V949D**	0	B	perturbation of hydrophobic environment by introduction of charged side-chain	83

Overall 213 point mutations of 160 residues. List of missense mutations retrieved from The Human Gene Mutation Database, HGMD (http://www.hgmd.cf.ac.uk/ac/index.php)

*ASA: percentage of accessible surface area of each residue calculated with the program Aereaimol[84]

B/E: buried/exposed: residues are considered to be buried if ASA is less than 10% and to be solvent exposed if the ASA value exceeds 40%.

Substrate	V_{MAX} (U mg^{-1})	k_{cat} (s^{-1})	K_M (mg ml^{-1})	k_{cat}/K_M (s^{-1} mg^{-1} ml)
**4NP-Glc**	4.3 ± 0.2	7.9 ± 0.3	1.9 ± 0.3	4.2
**Maltose**	97.1 ± 7.6	178.0 ± 13.9	14.0 ± 2.7	12.7
**Isomaltose**	3.3 ± 0.2	6.1 ± 0.4	16.5 ± 3.3	0.4
**Bovine glycogen**	27.3 ± 3.5	50.1 ± 6.4	69.6 ± 7.9	0.7
**Rabbit glycogen**	15.4 ± 2.3	28.2 ± 4.2	87.0 ± 23.3	0.3

Supplementary Table 2 | Substrate specificity of rhGAA. Steady state kinetic constants of rhGAA acting on 4Np-Glc, maltose, isomaltose, bovine glycogen and rabbit glycogen. Kinetic data were calculated as the average of at least two experiments and were plotted and refined with the program Prism 5.0 (GraphPad Software, San Diego California USA).

rhGAA forms	rhGAA precursor		rhGAA matured	
	-NAC	+NAC	-NAC	+NAC
Half point Temperature (Tm °C)	47.1 ± 0.4	60.3 ± 1.8	49.6 ± 0.3	60.3 ± 0.3
Stabilization (ΔTm °C)	-	13.2 ± 1.8	-	10.7 ± 0.6

Supplementary Table 3 | Analysis of the effect of the pharmacological chaperone NAC on the rhGAA precursor and the matured form. The table shows the melting temperatures and their relative shifts in absence and in presence of NAC. Thermal scans were performed in triplicate and fluorescence was normalized to the maximum value within each scan to obtain relative fluorescence. Melting temperatures were calculated according to Niesen *et al.*, 2007[4]. The standard deviations for each melting temperature were calculated from three replicates.

Stability parameter	Pharmacological chaperones		
	--	NAC (10 mM)	DNJ (0.1 mM)
**Half point temperature (Tm °C)**	68.9 ± 0.2	70.0 ± 0.2	78.5 ± 0.1
**Stabilization (ΔTm °C)**		1.1 ± 0.4	9.6 ± 0.3

Supplementary Table 4 | Analysis of the effect of pharmacological chaperones on α-glucosidase from rice. The Table shows the melting temperatures and their relative shifts in absence and in presence of the pharmacological chaperones NAC and DNJ. Thermal scans were performed in triplicate and fluorescence was normalized to the maximum value within each scan to obtain relative fluorescence. Melting temperatures were calculated according to Niesen *et al.*, 2007[4]. The standard deviations for each melting temperature were calculated from three replicates.

Supplementary References

1. Ren, L. *et al.* Structural insight into substrate specificity of human intestinal maltase-glucoamylase. *Protein. Cell.* **2,** 827-836 (2011).

2. Moreland, R. J. *et al.* Lysosomal acid alpha-glucosidase consists of four different peptides processed from a single chain precursor. *J. Biol. Chem.* **280,** 6780-6791 (2005).

3. Sim, L., Quezada-Calvillo, R., Sterchi, E. E., Nichols, B. L. & Rose, D. R. Human intestinal maltase-glucoamylase: crystal structure of the N-terminal catalytic subunit and basis of inhibition and substrate specificity. *J. Mol. Biol.* **375,** 782-792 (2008).

4. Niesen, F. H., Berglund, H. & Vedadi, M. The use of differential scanning fluorimetry to detect ligand interactions that promote protein stability. *Nat. Protoc.* **2,** 2212-2221 (2007).

5. Vivoli, M., Novak, H. R., Littlechild, J. A. & Harmer, N. J. Determination of protein-ligand interactions using differential scanning fluorimetry. *J. Vis. Exp.* 51809 (2014).

6. Hermans, M. M. *et al.* Twenty-two novel mutations in the lysosomal alpha-glucosidase gene (GAA) underscore the genotype-phenotype correlation in glycogen storage disease type II. *Hum. Mutat.* **23,** 47-56 (2004).

7. Kroos, M. *et al.* Update of the pompe disease mutation database with 60 novel GAA sequence variants and additional studies on the functional effect of 34 previously reported variants. *Hum. Mutat.* **33,** 1161-1165 (2012).

8. Wan, L. *et al.* Identification of eight novel mutations of the acid alpha-glucosidase gene causing the infantile or juvenile form of glycogen storage disease type II. *J. Neurol.* **255,** 831-838 (2008).

9. Nascimbeni, A. C., Fanin, M., Tasca, E. & Angelini, C. Molecular pathology and enzyme processing in various phenotypes of acid maltase deficiency. *Neurology.* **70,** 617-626 (2008).

10. Niu, D. M. *et al.* Novel human pathological mutations. Gene symbol: GAA. Disease: glycogen storage disease 2. *Hum. Genet.* **127,** 465 (2010).

11. Gort, L., Coll, M. J. & Chabás, A. Glycogen storage disease type II in Spanish patients: high frequency of c.1076-1G>C mutation. *Mol. Genet. Metab.* **92,** 183-187 (2007).

12. Chien, Y. H. *et al.* Later-onset Pompe disease: early detection and early treatment initiation enabled by newborn screening. *J. Pediatr.* **158,** 1023-1027.e1 (2011).

13. Kroos, M. *et al.* Update of the Pompe disease mutation database with 107 sequence variants and a format for severity rating. *Hum. Mutat.* **29,** E13-E26 (2008).

14. Pittis, M. G. *et al.* Molecular and functional characterization of eight novel GAA mutations in Italian infants with Pompe disease. *Hum. Mutat.* **29,** E27-E36 (2008).

15. Laforêt, P. *et al.* Juvenile and adult-onset acid maltase deficiency in France: genotype-phenotype correlation. *Neurology.* **55,** 1122-1128 (2000).

16. Oba-Shinjo, S. M. *et al.* Pompe disease in a Brazilian series: clinical and molecular analyses with identification of nine new mutations. *J. Neurol.* **256,** 1881-1890 (2009).

17. Fernandez-Hojas, R. *et al.* Identification of six novel mutations in the acid alpha-glucosidase gene in three Spanish patients with infantile onset glycogen storage disease type II (Pompe disease). *Neuromuscul. Disord.* **12,** 159-166 (2002).

18. Wittmann, J. *et al.* Newborn screening for lysosomal storage disorders in hungary. *JIMD. Rep.* **6,** 117-125 (2012).

19. Labrousse, P. *et al.* Genetic heterozygosity and pseudodeficiency in the Pompe disease newborn screening pilot program. *Mol. Genet. Metab.* **99,** 379-383 (2010).

20. Lin, H. Y. *et al.* Novel human pathological mutations. Gene symbol: GAA. Disease: glycogen storage disease 2. *Hum. Genet.* **127,** 464 (2010).

21. Pittis, M. G. *et al.* Identification of four novel mutations in the alpha glucosidase gene in five Italian patients with infantile onset glycogen storage disease type II. *Am. J. Med. Genet. A.* **121A,** 225-230 (2003).

22. Herzog, A. *et al.* A cross-sectional single-centre study on the spectrum of Pompe disease, German patients: molecular analysis of the GAA gene, manifestation and genotype-phenotype correlations. *Orphanet. J. Rare. Dis.* **7,** 35 (2012).

23. Anneser, J. M., Pongratz, D. E., Podskarbi, T., Shin, Y. S. & Schoser, B. G. Mutations in the acid alpha-glucosidase gene (M. Pompe) in a patient with an unusual phenotype. *Neurology.* **64,** 368-370 (2005).

24. Spada, M. *et al.* Screening for later-onset Pompe's disease in patients with paucisymptomatic hyperCKemia. *Mol. Genet. Metab.* **109,** 171-173 (2013).

25. Park, Y. E., Park, K. H., Lee, C. H., Kim, C. M. & Kim, D. S. Two new missense mutations of GAA in late onset glycogen storage disease type II. *J. Neurol. Sci.* **251,** 113-117 (2006).

26. Castro-Gago, M. *et al.* [Severe form of juvenile type II glycogenosis in a compound-heterozygous boy (Tyr-292--> Cys/Arg-854-->Stop)]. *Rev. Neurol.* **29,** 46-49 (1999).

27. Joshi, P. R. *et al.* Molecular diagnosis of German patients with late-onset glycogen storage disease type II. *J. Inherit. Metab. Dis.* **31 Suppl 2,** S261-S265 (2008).

28. Boerkoel, C. F. *et al.* Leaky splicing mutation in the acid maltase gene is associated with delayed onset of glycogenosis type II. *Am. J. Hum. Genet.* **56,** 887-897 (1995).

29. Bali, D. S. *et al.* Predicting cross-reactive immunological material (CRIM) status in Pompe disease using GAA mutations: lessons learned from 10 years of clinical laboratory testing experience. *Am. J. Med. Genet. C. Semin. Med. Genet.* **160C,** 40-49 (2012).

30. Kroos, M. A., van Leenen, D., Verbiest, J., Reuser, A. J. & Hermans, M. M. Glycogen storage disease type II: identification of a dinucleotide deletion and a common missense mutation in the lysosomal alpha-glucosidase gene. *Clin. Genet.* **53,** 379-382 (1998).

31. Zhong, N., Martiniuk, F., Tzall, S. & Hirschhorn, R. Identification of a missense mutation in one allele of a patient with Pompe disease, and use of endonuclease digestion of PCR-

amplified RNA to demonstrate lack of mRNA expression from the second allele. *Am. J. Hum. Genet.* **49,** 635-645 (1991).

32. Dou, W. *et al.* A novel missense mutation in the acid alpha-glucosidase gene causing the classic infantile form of Pompe disease. *Clin. Chim. Acta.* **374,** 145-146 (2006).

33. Manwaring, V. *et al.* Urine analysis of glucose tetrasaccharide by HPLC; a useful marker for the investigation of patients with Pompe and other glycogen storage diseases. *J. Inherit. Metab. Dis.* **35,** 311-316 (2012).

34. Lam, C. W. *et al.* Juvenile-onset glycogen storage disease type II with novel mutations in acid alpha-glucosidase gene. *Neurology.* **60,** 715-717 (2003).

35. Palmer, R. E. *et al.* Pompe disease (glycogen storage disease type II) in Argentineans: clinical manifestations and identification of 9 novel mutations. *Neuromuscul. Disord.* **17,** 16-22 (2007).

36. Niño, M. Y. *et al.* Identification and Functional Characterization of GAA Mutations in Colombian Patients Affected by Pompe Disease. *JIMD. Rep.* **7,** 39-48 (2013).

37. Van den Hout, J. M. *et al.* Long-term intravenous treatment of Pompe disease with recombinant human alpha-glucosidase from milk. *Pediatrics.* **113,** e448-e457 (2004).

38. Bali, D. S., Tolun, A. A., Goldstein, J. L., Dai, J. & Kishnani, P. S. Molecular analysis and protein processing in late-onset Pompe disease patients with low levels of acid α-glucosidase activity. *Muscle. Nerve.* **43,** 665-670 (2011).

39. Kishnani, P. S. *et al.* Chinese hamster ovary cell-derived recombinant human acid alpha-glucosidase in infantile-onset Pompe disease. *J. Pediatr.* **149,** 89-97 (2006).

40. Amartino, H. *et al.* Two clinical forms of glycogen-storage disease type II in two generations of the same family. *Clin. Genet.* **69,** 187-188 (2006).

41. Spada, M. *et al.* Spontaneous regression of hypertrophic cardiomyopathy in an infant with Pompe's disease. *Mol. Genet. Metab.* **107,** 763 (2012).

42. Dlamini, N. *et al.* Muscle MRI findings in siblings with juvenile-onset acid maltase deficiency (Pompe disease). *Neuromuscul. Disord.* **18,** 408-409 (2008).

43. Montalvo, A. L. *et al.* Mutation profile of the GAA gene in 40 Italian patients with late onset glycogen storage disease type II. *Hum. Mutat.* **27,** 999-1006 (2006).

44. Reuser, A. J. *et al.* Glycogenosis type II (acid maltase deficiency). *Muscle. Nerve. Suppl.* **3,** S61-S69 (1995).

45. Raben, N., Lee, E., Lee, L., Hirschhorn, R. & Plotz, P. H. Novel mutations in African American patients with glycogen storage disease Type II. Mutations in brief no. 209. Online. *Hum. Mutat.* **13,** 83-84 (1999).

46. McCready, M. E. *et al.* Development of a clinical assay for detection of GAA mutations and characterization of the GAA mutation spectrum in a Canadian cohort of individuals with glycogen storage disease, type II. *Mol. Genet. Metab.* **92,** 325-335 (2007).

47. Fujimoto, S. *et al.* A novel mutation of the GAA gene in a patient with adult-onset Pompe disease lacking a disease-specific pathology. *Intern. Med.* **52,** 2461-2464 (2013).

48. Huie, M. L., Hirschhorn, R., Chen, A. S., Martiniuk, F. & Zhong, N. Mutation at the catalytic site (M519V) in glycogen storage disease type II (Pompe disease). *Hum. Mutat.* **4,** 291-293 (1994).

49. Hermans, M. M. *et al.* Identification of a point mutation in the human lysosomal alpha-glucosidase gene causing infantile glycogenosis type II. *Biochem. Biophys. Res. Commun.* **179,** 919-926 (1991).

50. Müller-Felber, W. *et al.* Late onset Pompe disease: clinical and neurophysiological spectrum of 38 patients including long-term follow-up in 18 patients. *Neuromuscul. Disord.* **17,** 698-706 (2007).

51. Hermans, M. M. *et al.* The effect of a single base pair deletion (delta T525) and a C1634T missense mutation (pro545leu) on the expression of lysosomal alpha-glucosidase in patients with glycogen storage disease type II. *Hum. Mol. Genet.* **3,** 2213-2218 (1994).

52. Fernández, C., Legido, A., Jethva, R. & Marks, H. G. Correction of a short cardiac PR interval in a 12-year-old girl with late-onset Pompe disease following enzyme replacement therapy. *Genet. Med.* **14,** 757-758 (2012).

53. Raben, N., Plotz, P. & Byrne, B. J. Acid alpha-glucosidase deficiency (glycogenosis type II, Pompe disease). *Curr. Mol. Med.* **2,** 145-166 (2002).

54. Loureiro Neves, F. *et al.* [Juvenile Pompe disease: retrospective clinical study]. *Acta. Med. Port.* **26,** 361-370 (2013).

55. Alcántara-Ortigoza, M. A. *et al.* Screening of late-onset Pompe disease in a sample of Mexican patients with myopathies of unknown etiology: identification of a novel mutation in the acid alpha-glucosidase gene. *J. Child. Neurol.* **25,** 1034-1037 (2010).

56. Hermans, M. M. *et al.* Glycogen Storage Disease type II: genetic and biochemical analysis of novel mutations in infantile patients from Turkish ancestry. *Hum. Mutat.* **11,** 209-215 (1998).

57. Sharma, M. C. *et al.* Delayed or late-onset type II glycogenosis with globular inclusions. *Acta. Neuropathol.* **110,** 151-157 (2005).

58. Pipo, J. R. *et al.* New GAA mutations in Japanese patients with GSDII (Pompe disease). *Pediatr. Neurol.* **29,** 284-287 (2003).

59. Tsujino, S. *et al.* Frequent mutations in Japanese patients with acid maltase deficiency. *Neuromuscul. Disord.* **10,** 599-603 (2000).

60. Ko, T. M. *et al.* Molecular genetic study of Pompe disease in Chinese patients in Taiwan. *Hum. Mutat.* **13,** 380-384 (1999).

61. Muraoka, T. *et al.* Novel mutations in the gene encoding acid α-1,4-glucosidase in a patient with late-onset glycogen storage disease type II (Pompe disease) with impaired intelligence. *Intern. Med.* **50,** 2987-2991 (2011).

62. Kroos, M. *et al.* Seven cases of Pompe disease from Greece. *J. Inherit. Metab. Dis.* **29,** 556-563 (2006).

63. Vorgerd, M., Burwinkel, B., Reichmann, H., Malin, J. -P. & Kilimann, M. W. Adult-onset glycogen storage disease type II: phenotypic and allelic heterogeneity in German patients. *Neurogenetics.* **1,** 205-211 (1998).

64. Scott, C. R. *et al.* Identification of infants at risk for developing Fabry, Pompe, or mucopolysaccharidosis-I from newborn blood spots by tandem mass spectrometry. *J. Pediatr.* **163,** 498-503 (2013).

65. Hermans, M. M., Kroos, M. A., de Graaff, E., Oostra, B. A. & Reuser, A. J. Two mutations affecting the transport and maturation of lysosomal alpha-glucosidase in an adult case of glycogen storage disease type II. *Hum. Mutat.* **2,** 268-273 (1993).

66. Huie, M. L. *et al.* Glycogen storage disease type II: identification of four novel missense mutations (D645N, G648S, R672W, R672Q) and two insertions/deletions in the acid alpha-glucosidase locus of patients of differing phenotype. *Biochem. Biophys. Res. Commun.* **244,** 921-927 (1998).

67. Hermans, M. M. *et al.* The conservative substitution Asp-645-->Glu in lysosomal alpha-glucosidase affects transport and phosphorylation of the enzyme in an adult patient with glycogen-storage disease type II. *Biochem. J.* **289** , 687-693 (1993).

68. Lin, C. Y. & Shieh, J. J. Identification of a de novo point mutation resulting in infantile form of Pompe's disease. *Biochem. Biophys. Res. Commun.* **208,** 886-893 (1995).

69. Huie, M. L., Chen, A. S., Brooks, S. S., Grix, A. & Hirschhorn, R. A de novo 13 nt deletion, a newly identified C647W missense mutation and a deletion of exon 18 in infantile onset glycogen storage disease type II (GSDII). *Hum. Mol. Genet.* **3,** 1081-1087 (1994).

70. Terzis, G. *et al.* Effects of exercise training during infusion on late-onset Pompe disease patients receiving enzyme replacement therapy. *Mol. Genet. Metab.* **107,** 669-673 (2012).

71. Kroos, M. A. *et al.* p.[G576S; E689K]: pathogenic combination or polymorphism in Pompe disease? *Eur. J. Hum. Genet.* **16,** 875-879 (2008).

72. Montalvo, A. L. *et al.* Glycogenosis type II: identification and expression of three novel mutations in the acid alpha-glucosidase gene causing the infantile form of the disease. *Mol. Genet. Metab.* **81,** 203-208 (2004).

73. Qiu, J. J., Wei, M., Zhang, W. M. & Shi, H. P. [Clinical and molecular genetic study on two patients of the juvenile form of Pompe disease in China]. *Zhonghua. Er. Ke. Za. Zhi.* **45,** 760-764 (2007).

74. Cho, A. *et al.* Infantile Pompe disease: clinical and genetic characteristics with an experience of enzyme replacement therapy. *J. Child. Neurol.* **27,** 319-324 (2012).

75. Ebrahim, H. Y., Baker, R. J., Mehta, A. B. & Hughes, D. A. Functional analysis of variant lysosomal acid glycosidases of Anderson-Fabry and Pompe disease in a human embryonic kidney epithelial cell line (HEK 293 T). *J. Inherit. Metab. Dis.* **35,** 325-334 (2012).

76. Sampaolo, S. *et al.* Distinct disease phenotypes linked to different combinations of GAA mutations in a large late-onset GSDII sibship. *Orphanet. J. Rare. Dis.* **8,** 159 (2013).

77. Prater, S. N. *et al.* The emerging phenotype of long-term survivors with infantile Pompe disease. *Genet. Med.* **14,** 800-810 (2012).

78. El-Gharbawy, A. H. *et al.* Expanding the clinical spectrum of late-onset Pompe disease: dilated arteriopathy involving the thoracic aorta, a novel vascular phenotype uncovered. *Mol. Genet. Metab.* **103,** 362-366 (2011).

79. van der Beek, N. A. *et al.* Cardiac evaluation in children and adults with Pompe disease sharing the common c.-32-13T>G genotype rarely reveals abnormalities. *J. Neurol. Sci.* **275,** 46-50 (2008).

80. Musumeci, O. *et al.* Auditory system involvement in late onset Pompe disease: a study of 20 Italian patients. *Mol. Genet. Metab.* **107,** 480-484 (2012).

81. van den Hout, H. M. *et al.* The natural course of infantile Pompe's disease: 20 original cases compared with 133 cases from the literature. *Pediatrics.* **112,** 332-340 (2003).

82. Kroos, M. A. *et al.* A case of childhood Pompe disease demonstrating phenotypic variability of p.Asp645Asn. *Neuromuscul. Disord.* **14,** 371-374 (2004).

83. Becker, J. A. *et al.* The African origin of the common mutation in African American patients with glycogen-storage disease type II. *Am. J. Hum. Genet.* **62,** 991-994 (1998).

84. Lee, B. & Richards, F. M. The interpretation of protein structures: estimation of static accessibility. *J. Mol. Biol.* **55,** 379-400 (1971).
